# The effect of ethnicity on prescribing practice and treatment outcome in inpatients suffering from schizophrenia in Greece

**DOI:** 10.1186/1471-244X-11-66

**Published:** 2011-04-20

**Authors:** Athanassios Douzenis, Athanassios Apostolopoulos, Dionisios Seretis, Emmanouil N Rizos, Christos Christodoulou, Lefteris Lykouras

**Affiliations:** 1 Second Psychiatry Department, Athens University Medical School, Attikon General Hospital, 1 Rimini st Athens 12462, Greece

## Abstract

**Background:**

No studies have been conducted in Greece with the aim of investigating the influence of ethnicity on the prescribing and treatment outcome of voluntarily admitted inpatients. Most studies conducted in the UK and the US, both on inpatients and outpatients, focus on the dosage of antipsychotics for schizophrenic patients and many suffer from significant methodological limitations. Using a simple design, we aimed to assess negative ethnic bias in psychotropic medication prescribing by comparing discrepancies in use between native and non-native psychiatric inpatients. We also aimed to compare differences in treatment outcome between the two groups.

**Methods:**

In this retrospective study, the prescribing of medication was compared between 90 Greek and 63 non-Greek inpatients which were consecutively admitted into the emergency department of a hospital covering Athens, the capital of Greece. Participants suferred from schizophrenia and other psychotic disorders. Overall, groups were compared with regard to 12 outcomes, six related to prescribing and six related to treatment outcome as assesed by standardised psychometric tools.

**Results:**

No difference between the two ethnic groups was found in terms of improvement in treatment as measured by GAF and BPRS-E. Polypharmacy, use of first generation antipsychotics, second generation antipsychotics and use of mood stabilizers were not found to be associated with ethnicity. However, non-Greeks were less likely to receive SSRIs-SNRIs and more likely to receive benzodiazepines.

**Conclusions:**

Our study found limited evidence for ethnic bias. The stronger indication for racial bias was found in benzodiazepine prescribing. We discuss alternative explanations and give arguments calling for future research that will focus on disorders other than schizophrenia and studying non-inpatient populations.

## Background

Ethnicity as a form of clinical bias in psychiatric contexts has long been debated and researched. The effect of ethnicity in clinical practice has been investigated in reference to diagnosis [1] and treatment (i.e. prescribing practices [2], psychotherapy outcome [3]) - for both in-patients as well as outpatients. More recently several studies have identified clinical bias and heightened concern has been raised, calling for more extensive research on institutional racism, and the training of staff to address disparities in both admission and use of mental health services. It has been shown, for example, that ethnic minority group members tend to be perceived as having more psychopathological traits compared to other population groups and are more likely to be admitted to psychiatric wards [4].

However, findings are relatively inconsistent as several other studies have identified no such biases among patients of different ethnicities [5-7]. Research in this area is extensive and the bulk of studies have focused on patients with schizophrenia [8-11]. In cases where institutional bias was identified in the prescribing patterns in these studies, most researchers focused on antipsychotic medication differences (i.e. first generation antipsychotics -FGAs- versus second generation antipsychotics -SGAs) rather than looking at other categories of drugs. Further limitations with antipsychotic drug prescribing arise from the inherent methodological difficulties within empirical work in this field of inquiry. For instance, Yorston and Pinney [12] outline the problems of using a standard, and commonly used method of measuring total antipsychotic dose in terms of chlorpromazine equivalents. Moreover, while institutional bias and ethnicity have been investigated thoroughly with regard to primary care of psychiatric patients, there are fewer studies on psychiatric in-patients.

Additionally, the vast majority of the studies come from the US and the UK [13] and to a lesser extent from other, developed 'western countries'. Thus, whereas issues such as ethnicity and race have been debated for a long time in these countries, other countries less economically developed have not contributed to this debate. To our knowledge there have been no studies in Greece regarding race and ethnicity affecting psychiatric prescribing practices. This is not surprising as Greece has been up until recently a fairly homogeneous country. This has changed and since the late seventies Greece has received a large amount of immigrants. Initially these were of Greek origins that returned from countries of the 'Eastern bloc '. Later, immigrants from the Balkan states (mainly Albania) arrived. They were followed by immigrants of Kurdish, Afghan and African origin.

In this first study of assessing ethnicity as a form of clinical bias in voluntarily admitted inpatients' treatment, our aim was to investigate for possible differences with regard to prescribing practice, focusing on use rather than dosage. Thus, the design of the study aimed at being simple in order to minimize the possibility that our findings were compromised by methodological limitations inherent in these type of studies. We aimed to investigate for negative ethnic bias across native and non-native inpatients that fulfilled the DSM criteria for schizophrenia and other psychotic disorders (schizoaffective and delusional disorder). Rather than focusing only on antipsychotic medication, we tested for differences in prescribing for mood stabilizers, SSRIs-SNRIs, benzodiazepines, drugs which are often administered to schizophrenic patients. Additionally, we aimed to compare treatment outcome across ethnic groups as assessed by two commonly used scales General Assessment of Functioning (GAF) and the Brief Psychiatric Rating Scale (BPRS-E).

## Methods

The study was conducted at the Second Psychiatric Clinic of Athens University Medical School in Attikon General Hospital which had recently opened. This unit does not accept involuntary admissions. Our sample comprised of 153 in-patients admitted consecutively via the emergency department of the hospital from April 2008 to December 2009. The emergency department is on call twice a week (Mondays and Thursdays), covering the area of Attica, with another large psychiatric hospital which assesses requests for involuntary admissions. Attica is the most densely populated geographical area in Greece, including Athens where half of the residents in Greece live. The age of patients ranged from 19 to 59 years old; 81 were males and 72 females. They were designated as Greek (N = 90) or non-Greek (N = 63) if both their parents were either Greek or non-Greek respectively. Patients from mixed-ethnicity marriages were excluded from the study. All patients fulfilled the DSM-IV-TR criteria for schizophrenia and other psychotic disorders (schizoaffective and delusional). Admission of patients was made on a voluntary basis alone. No restraint, enforced medication or seclusion was used for any of the patients.

Based on the relevant literature we checked for several confounding variables, including length of stay, age, legal status upon admission, substance misuse upon admission, prior substance misuse and smoking status on admission.

Each patient received an initial diagnosis and admission approval by the two psychiatrists responsible for the emergency department on the day of admission. Overall, 18 trained psychiatrists participated in this study. The on call psychiatrists were aware that their clinical judgments would be used in a study about medication prescribing. They were blind to the fact that the study explored ethnic bias in clinical practice. All psychiatrists were of Greek nationality with both parents Greek.

The social and psychological functioning of patients was assessed by the Global Assessment of Functioning (GAF) scale. GAF is widely used in mental health practice and has established good validity [14,15]. Moreover, because GAF is meant to assess patients' functioning and not their psychiatric symptoms, the brief psychiatric rating scale-extended version (BPRS-E) [16] was also used to assess symptom severity on admission and discharge. The BPRS comprises of 24 symptom constructs, each rated on a 7-point scale of severity. This rating scale been used extensively with populations with severe and persistent mental problems and has established sound reliability and validity

Medication (types and doses of drugs) upon admission was determined by the two psychiatrists that had agreed to admit each patient. GAF and BPRS-E ratings upon admission were conducted by the same two psychiatrists. Medication on discharge as well as GAF and BPRS ratings on discharge were determined by the responsible attending psychiatrist which was one of the two psychiatrists that had admitted the patient.

Ethical approval for this research was granted by the Attikon University General Hospital ethics Committee and consequently by the Attikon General Hospital Scientific Committee. Written informed consent was obtained by the patients. Overall, data were gathered by the medical and nursing files of the Department as well as the attending psychiatrist and nursing staff.

Prescribing practice was compared between the groups with reference to 5 factors:

1. Polypharmacy was defined as the concurrent treatment with more than one psychiatric medication (as opposed to treatment with one or more antipsychotic agents [17] employed in most other studies). Thus, in our study it consisted simply in the sum, in absolute number, (rather than rated in doses, usually sum of individual % of the maximum dose allowed) of the different drugs given in each of the following categories: FGAs, SGAs, mood stabilizers, SSRIs-SNRIs and benzodiazepines. If in one category more than one drug was given, this would also be counted (two mood stabilizers, one SGA and two different benzodiazepines would make up 5 in our score). In Greece, antipsychotic polypharmacy is very widespread and is a product of poor yet well-meant and standard practice. Thus we figured that elevated use of antipsychotic polypharmacy in non-Greeks would be difficult to associate with intended racial behavior. The latter might be better assessed by the measure adopted in our study, especially as Greek psychiatrists are well aware of the negative short and long terms consequences of polypharmacy,

2. Use of FGAs (yes/no)

3. Use of SGAs (yes/no)

4. Use of mood stabilizers (yes/no)

5. Use of SSRI-SNRI. (Yes/no)

6. Use of benzodiazepines (Yes/no)

Thus, we compared six outcomes related to prescribing and six scale outcomes; differences in BPRS-E and GAF on admission, on discharge and on improvement as rated by each scale (that consisted of the mean differences in ratings-discharge mean minus admission mean for each ethnic group).

In total we tested for twelve two-tailed experimental hypotheses. Based on the literature we expected to find some evidence of negative ethnic bias towards the ethnic minority group members in terms of one or more categories tested.

## Results

Table 1 shows the countries of origin of the non-Greeks admitted. The vast majority comprises of patients of Albanian origin, 50, 79% of all non-Greeks.

**Table 1:** Countries of origin among non-Greek Patients

Country of origin	Number of patients
**Albania**	32
**Romania**	4
**Russia**	4
**Afghanistan**	3
**Pakistan**	3
**Bulgaria**	3
**Moldavia**	3
**Poland**	2
**Kazakhstan**	2
**Tunisia**	2
**Nigeria**	2
**Germany**	1
**Cuba**	1
**South Africa**	1

Table 2 shows the diagnoses and chronicity across the two groups. Pearson Chi square tests were non-significant for both diagnostic category x2(2) = 1,408 p > 0.05 and chronicity x^2 ^(1) = 0,217 p > 0.05

**Table 2:** Diagnoses and chronicity across the two ethnic groups

		Participant's Ethnicity		Total
		Greek	Foreign	
**Participants' ** **diagnosis**	Schizophrenia	69	51	120
		76,70%	81,00%	78,40%
	Delusional	12	9	21
		13,30%	14,30%	13,70%
	Schizoaffective	9	3	12
		10,00%	4,80%	7,80%
**Chronicity**	First episode	48	36	84
		53,30%	57,10%	54,90%
	Chronic patient	42	27	69
		46,70%	42,90%	45,10%

Table 3 shows the demographic and clinical variables of the study.

**Table 3:** Sociodemographic and clinical variables across the two ethnic groups

	Greeks		Non-Greeks		2-sided asymptotic significance
	
	90 58,8% of sample		63 41,2% of sample		
Gender: N and % within ethnic group	42 m (46, 7%),	48 f (53, 3%)	39 m (61, 9%)	24 f (38, 1%)	Pearson Chi-Square Sig. 0,063(ns)

Age	Mdn 36 Mean 36.36 SD 10.46 Range19-55		Mdn 34 Mean 35.04 SD10.69 Range:19-59		Mann Whitney Sig. 0,285(ns)

Smoking status: N and % within ethnic group	81 yes (90%)	9 no (10%)	54 yes (85,7%)	9 no (14,3%)	Pearson Chi-Square Sig. 0,418(ns)

Forensic status upon admission N and %within ethnic group	6 yes (6,7%)	84 no (93,3%)	0 yes (0, 0%)	63 no (100, 0%)	**Pearson Chi-Square** **Sig. 0,037**

Substance abuse on admission N and % within ethnic group	9 yes (10,0%)	81 no (90,0%)	12 yes (19,0%)	51 no (81,0%)	Pearson Chi-Square Sig. 0,109(ns)

History of substance abuse and % within ethnic group	21 yes (23, 3%)	69 no (76,7%)	15 yes (23, 8%)	48 no (76, 2%)	Pearson Chi-Square Sig. 0,946(ns)

Length of stay	Mdn 19 Mean20.33 SD 8.46 Range 8-40		Mdn18 Mean 21.81 SD 14.26 Range 5-75		Mann Whitney Adj. Sig. 0,894(ns)

BPRS improvement	Mdn38 Mean 40,16 SD 11,78		Mdn 41 Mean 41,66 SD10,92		Mann Whitney Adj.Sig. 0,187(ns)

BPRS on admission	Mdn 71 Mean 71,97, SD12,15		Mdn 70 Mean 72,95 SD 12,21		Mann Whitney Adj.Sig. 0,385(ns)

BPRS on discharge	Mdn 30, 50 Mean, 31,80 SD 4,24		Mdn 29 Mean 31,29 SD 6,86		Mann Whitney Adj.Sig. 0,033(ns)

GAF improvement	Mdn 25 Mean 26 SD 7,72		Mdn 30 Mean 26,90 SD 10,25		Mann Whitney Adj.Sig. 0,352(ns)

GAF on admission	Mdn 40 Mean 41 SD 8,04		Mdn 40 Mean 41,67 SD 10,12		Mann Whitney Adj.Sig. 0,760(ns)

GAF on discharge	Mdn 70 Mean 67 SD 5,12		Mdn 70 Mean 68,57 SD 6,8		Mann Whitney Adj.Sig. 0,036(ns)

Polypharmacy on admission	Mdn 4 Mean 3,80 SD 1,59		Mdn 4 Mean 3,95 SD 1,22		Mann Whitney Adj.Sig.0,634(ns)

FGAs on admission N and % within ethnic group	57 yes (63, 3%)	33 no (36, 7%)	45 yes (71,4%)	18 no (28,6%)	Pearson Chi-Square Adj.Sig.0,296(ns)

SGAs on admission N and % within ethnic group	81 yes (90,0%)	9 no (10,0%)	63 yes (100,0)	0 no (0%)	Pearson Chi-Square Adj. Sig. 0,011(ns)

SSRIs-SNRIs on admission N and % within ethnic group	39 yes (43,3%)	51 no (56,7%)	3 yes (4,8%)	60 no (95,2%)	**Pearson Chi-Square** **Sig. 0,000** **Fisher's exact** **Sig. 0,000**

Mood stabilizers on admission and % within ethnic group	24 yes (26,7%)	66 no (73,3%)	12 yes (19,0%)	51 no (81,0%)	Pearson Chi-Square Adj. Sig. 0,274(ns)

Benzodiazepines on admission and % within ethnic group	53 yes (58, 9%)	37 no (41,1%)	57 yes (90,5%)	6 no (9,5%)	**Pearson Chi-Square** **Sig. 0,000** **Fisher's exact** **Sig. 0,000**

Mann-Whitney tests, and chi-square tests gave no significant differences between the groups in smoking status, age, gender, history of substance misuse, substance misuse upon admission. All non-Greeks had no forensic status upon admission compared to 93,3% of Greeks having no forensic status upon admission x^2 ^(1) = 4,371 p < 0.05.

For the remaining of the variables the significance level was adjusted for multiple comparisons by the Holm-Bonferroni method.

Mann-Whitney tests gave no signficant differences in: BPRS on admission, BPRS on discharge, BPRS improvement, GAF on admission, GAF on discharge, GAF improvement ratings, length of stay and polypharmacy across ethnic groups.

Pearson chi-square tests gave no significant difference in the use of mood stabilizers, use of FGAs or SGAs across ethnic groups.

Pearson chi square tests gave significant differences across ethnic groups in: use of SSRIs-SNRIs between the groups x^2^(1) = 27,684 p = 0,00 and use of benzodiazepines x^2^(1) = 18,79, p = 0,00. Correcting for the non-significant gender imbalance between the two ethnic groups we still obtained: for use of SSRIs-SNRIs x^2^(1) = 32,942 p = 0,00 (and Fisher's exact p = 0,00), the phi 0,464, with 54,4% of Greeks non receiving SSRIs-SNRIs as opposed to 96,8% of non-Greeks; for use of benzodiazepines x2(1) = 17,277 p = 0,00 (and Fisher's exact p = 0,00), the phi 0,336, with 42,2% of Greeks not receiving benzodiazepines as opposed to only 11,1 of non-Greeks. In order to attempt to explain the big differences between SSRI-SNRI and benzodiazepine use between Greek and ethnic minority patients, we carried out Mann-Whitney tests for the depression, anxiety, motor hyperactivity and tension items of the BPRS-E on admission. In these four items of the scale, no significant difference between the groups was found (with alpha level both unadjusted and adjusted by the Holm-Bonferroni procedure).

## Discussion

Prescribing, in terms of FGAs and SGAs, was not found to be influenced by ethnicity. These findings are in contrast to studies which have revealed lower use of SGAs among members of American ethnic minority groups within outpatients suffering from schizophrenia [18-20], but in agreement with the results of Connolly and Taylor [5] who focused on in-patients' use of SGAs. Our findings may suggest that the relationship between in-patient care and ethnicity might be subject to variables different than the ones found in outpatient care.

Polypharmacy was not associated with ethnicity. Connolly and Taylor [5] report no differences in antipsychotic polypharmacy with the exception of one centre only. Covell et al [17] also report no association between polypharmacy and ethnicity. Our finding however cannot be compared to the latter as a different definition and measure of polypharmacy was used. Nevertheless, our rate of polypharmacy will still be associated with a number of adverse consequences for patients, discussed in Covell et al. This findings are of significance as polypharmacy was in the past measured primarily among outpatients

Based on the above, it could be argued that Greek psychiatrists are not influenced in their prescribing of antipsychotics by the patient's ethnicity. Greeks themselves have both been immigrants in recent history and experienced discrimination and prejudice. This history could act as a sensitizing factor. Moreover, the fact that Greece has only recently received immigrants can lead us to the assumption that racial stereotypes have not been developed as yet. Another explanation is that most of the immigrants (esp. the ones from the Balkan states) speak very good Greek and lack of communication difficulties (and skin colour) can account for the lack of racial bias in some of the patients.

On the other hand, prescribing in terms of SSRIs during hospitalization was influenced by inpatient ethnicity. Ethnic minority group members were less likely to receive SSRIs. One explanation is that, at the time of the study generic SSRIs and SNRIs were not widely available in Greece and their price was not as low as it was in the rest of Europe. It is only since 2010 that all medication prices have been substantially reduced and the state has started putting pressure on doctors to change their prescribing habits. Contrary to the SSRIs generic SGAs were available at the time of the study and were used. On the other hand, one cannot dismiss the argument that Greek doctors SSRIs and SNRIs prescribing for patients with schizophrenia, schizoaffective and delusional disorder implies a long term treatment plan and the absence of it might be an indication of racist bias. Ethnicity can influence psychiatric practice in subtle ways [21]. In addition, the significant lack of studies in terms of SSRI prescribing in terms of inpatients suggests that further research should be undertaken. The same applies with regard to mood stabilizers. A recent survey found longitudinal differences in antimanic medication use for blacks compared with whites [22]. However, significant absence of previous studies investigating mood stabilizers' prescribing does not allow comparison and thus generalization of findings in this field.

The large difference in benzodiazepines prescribing for ethnic minority patients cannot be explained by differences in the BPRS-E items of anxiety, hyperactivity and tension at baseline. The only plausible explanation for this finding is that Greek doctors and nurses perceived these patients as being more agitated and dangerous and felt the need to sedate them. Research on racial influences on benzodiazepines prescribing is very limited on schizophrenia inpatients as an indication of racist bias [23]

Our study design had limitations. Firstly, prescribing doctors were aware that the study protocol of the authors would relate to prescribing practice. We expected that this might have been at least in part offset by the fact that all admitting psychiatrists have their prescriptions reviewed regularly during the ward rounds. In this respect, their prescribing is always judged irrespective of any research. However, the fact that psychiatrists might have been more conscious of their treatment options remains a weakness and the chance that this might have biased the results cannot be dismissed altogether. It is worth emphasizing that the prescribing doctors were blind to the fact that the study explored ethnic bias. Secondly, the group members were not matched for social class or income. This could have biased the results. It has convincingly been argued that over representation of certain diagnoses as well as increased rates of admission among ethnic minority groups may not be a matter of institutional racism per se or a matter of overpathologizing bias (i.e. due to misdiagnosis owed to the misconception of cultural-specific behavioral cues, as fulfilled diagnostic criteria). Instead, it may reflect adverse social influences leading to increased levels of certain mental conditions in members of minorities [24-26]. McGuire and Miranda [27] provide a very good discussion on a number of factors which produce clinician bias and stereotyping, leading to disparities in mental health practice. Furthermore, our the generalisability of our study is, to an extent, limited by the fact that the groups compared were all in-patients in a single urban hospital. It is also worth noting that non-Greeks were under-represented in this study.

## Conclusions

This was a naturalistic study of psychotropic medication prescribing in a newly established Psychiatric Clinic in a General Hospital. We found limited evidence for ethnic bias in the treatment given. The stronger indication for racial bias was found in benzodiazepine prescribing. A study which investigated ethnic bias in patients involuntarily admitted [28] in Greece, Italy and the UK, revealed a far more controlling and restraining attitude from Greek psychiatric institutions when compared to Italy and the UK. Our literature review for this study underlined the absence of studies assessing prescribing patterns for use for antidepressants (SSRIs), mood stabilizers, and in particular for benzodiazepines. This is of interest, again underlining the fact that most studies on ethnic bias are related to schizophrenia in the UK and the US. Racial and ethnic bias though can be powerfully expressed in the treatment (or neglect) for other debilitating and distressing psychiatric disorders. Ethnicity can influence psychiatric practice in many, sometimes very subtle ways and future larger-scale studies focusing on inpatients and outpatients from less developed economically countries are needed. In particular, the overall pattern of the findings further suggest that there is an urgent need for greater-scale research to investigate racial and/or overpathologizing bias towards ethnic minority inpatients. Differences between studies might result as of factors that are expected to be different within outpatients and inpatients.

## Competing interests

The authors declare that they have no competing interests.

## Authors' contributions

AD designed the study and wrote part of the paper, and corrected the final draft

AA collected the data and wrote part of the paper. DS did the statistical analysis and wrote part of the paper. ENR and CC contributed towards the design of the study LL had the overall overseeing of the research read and made corrections in the manuscript.

All authors have read and approved the manuscript

## Pre-publication history

The pre-publication history for this paper can be accessed here:

http://www.biomedcentral.com/1471-244X/11/66/prepub
